# Appetite and Satiety Effects of the Acute and Regular Consumption of Green Coffee Phenols and Green Coffee Phenol/Oat β-Glucan Nutraceuticals in Subjects with Overweight and Obesity

**DOI:** 10.3390/foods10112511

**Published:** 2021-10-20

**Authors:** Mónica Redondo-Puente, Raquel Mateos, Miguel A. Seguido, Joaquín García-Cordero, Susana González, Rosa M. Tarradas, Laura Bravo-Clemente, Beatriz Sarriá

**Affiliations:** Department of Metabolism and Nutrition, Institute of Food Science, Technology and Nutrition (ICTAN-CSIC), Spanish National Research Council (CSIC), José Antonio Nováis 10, 28040 Madrid, Spain; m.redondo@ictan.csic.es (M.R.-P.); raquel.mateos@ictan.csic.es (R.M.); m.seguido@ictan.csic.es (M.A.S.); yoaquin1995@gmail.com (J.G.-C.); s.gonzalez@ictan.csic.es (S.G.); rosa.tarradas.valero@gmail.com (R.M.T.); lbravo@ictan.csic.es (L.B.-C.)

**Keywords:** green coffee, beta-glucan, nutraceutical, polyphenols, satiety, ghrelin, leptin, overweight

## Abstract

Green coffee has weight management properties, yet its effects on appetite and satiety remain unclear as few, mainly acute, studies perform objective measurements. Therefore, the influence on appetite/satiety of acute and regular consumption of two nutraceuticals, a decaffeinated green coffee phenolic extract (GC) alone or combined with oat β-glucans (GC/BG), with known satiating properties, has been analysed subjectively using visual analog scales (VAS) and objectively measuring actual food intake and postprandial appetite and satiety hormones. A randomised, cross-over, blind trial was carried out in 29 overweight volunteers who consumed GC or GC/BG twice a day for 8 weeks. After acute (day = 0) and regular consumption (day = 56) of the nutraceuticals, satiety was measured at 30, 60, 90, 150, and 210 min, as well as food intake at breakfast (30 min) and lunch (300 min). Additionally, in a subgroup of participants (*n* = 9), cholecystokinin, peptide-YY, glucagon-like-peptide-1, ghrelin and leptin concentrations were analysed in blood samples taken at the same time-points. According to VAS results, GC/BG reduced hunger more efficiently than GC. However, there were no statistically significant differences in food intake. Comparing the effects of the acute consumption of GC/BG and GC, leptin concentration at 150 min was higher after GC/BG intake vs. GC. Moreover, when comparing the effects of regularly consuming the two nutraceuticals, maximum ghrelin level decreased with GC/BG vs. GC. In conclusion, acute and regular effects of the nutraceuticals on appetite/satiety differed, and subjective and objective results partially agreed; GC/BG may reduce hunger more efficiently than GC.

## 1. Introduction

Obesity is a major public health concern. According to the World Health Organization (WHO), in 2016 more than 1.9 billion adults were overweight and 650 million were categorized as obese. Obesity is a known risk factor for numerous health problems, such as type 2 diabetes, cardiovascular diseases, and some forms of cancer [1]. This disease is often treated through strategies consisting of increasing exercise and restricting food intake, which are difficult to follow in the long-term. Therefore, alternative ways to help lose weight need to be found. In this context, dietary supplements or nutraceuticals may be an interesting aid for the prevention of overweight, obesity and their associated metabolic disorders [2]. According to Stuby et al. [3], bioactive phytochemicals in food supplements are a promising approach to improve patients’ adherence to reducing food and caloric intake.

Dietary supplements or nutraceuticals often consist of natural bioactive compounds with health-promoting properties. There are numerous nutraceuticals for weight loss based on polyphenol (PP) rich plant extracts [4,5]. Regarding their efficacy in suppressing appetite/hunger and/or increasing satiety, the most commonly tested plant extracts derive from *Camellia sinensis* (green tea), *Capsicum annuum* (chili), and *Coffea* species [3]. Besides enhancing metabolic rate, these naturally derived food supplements may also suppress energy intake [6]. However, none of the extracts have shown a consistent positive treatment effect in the aforementioned parameters. Concerning coffee, chlorogenic acid (CGA), the major phenolic compound in coffee, could play an important role as shown in animal experiments, having antiobesity effects by inducing a decrease in body weight and visceral fat mass, while caffeic acid, also present in coffee in important amounts, had weaker effects [7]. In humans, in a recent systematic review and dose–response meta-analysis of randomised clinical trials, it was concluded that green coffee extract supplementation has a beneficial effect on body weight, body mass index (BMI) and waist circumference (WC), and thus was pointed out as a potentially adequate treatment for obesity [8]. It is well known that some of the mechanisms responsible for the antiobesity effects of green coffee are the regulation of adipogenesis, changes in transcription factors as well as the expression and levels of genes and proteins associated with lipid metabolism in white adipose tissue and liver [9]. However, the effects of GC on appetite/satiety have been less explored, and so far, controversial results have been obtained.

Broad evidence shows that energy intake is controlled not only by the central nervous system but also by hormones secreted by the adipose tissue, that produce long-term regulation, and by gastrointestinal proteins that control satiety during meals. In this context, outstanding cholecystokinin (CCK), peptide tyrosine–tyrosine (PYY), and glucagon like peptide-1 (GLP-1), that are secreted by the gastrointestinal tract after food intake, along with leptin secreted by the adipose tissue, all induce satiety as recently reviewed in Stuby et al. [3]. In contrast, ghrelin, also secreted at the gastrointestinal tract, has appetite-activating capacity and decreases rapidly after ingestion of food, progressively rising before the next meal [10]. These peripheral signals eventually reach the central nervous system via neural pathways (via the vagus nerve) or humoral pathways (as endocrine secretions into the bloodstream) where they activate different brain regions, inducing satiety or energy intake [11,12]. It seems that CGA could reduce hunger by increasing PYY levels as well as stimulating secretion of the incretin hormone GLP-1 [13,14]. PYY and GLP-1, have similar effects, as PYY may lower food intake in humans and animals and also reduce body weight gain in animals [15], whereas intravenous administration of GLP-1 has been found to acutely decrease food intake, and after long-term administration cause weight loss in humans [16]. However, there is controversy regarding acute effects of coffee on GLP-1; whereas Johnston et al. [17] found that decaffeinated coffee yielded higher levels of GLP-1 than placebo, Gavrieli et al. [18] described that decaffeinated coffee did not affect GLP-1 levels in plasma. In a long-term intervention carried out in humans, a decaffeinated green coffee bean extract was suggested to contribute to appetite control, which was assessed by means of a simplified nutritional appetite questionnaire [19]. In agreement, Sarriá et al. [20] pointed to a green/roasted coffee blend controlling weight in normoweight subjects through ghrelin regulation, as ghrelin concentration was inversely related to body weight, and also WC and WC/hip ratios. Moreover, it seems that this hormone plays a role in energy adaptation, as ghrelin levels decrease in humans with obesity and metabolic syndrome, and increase during weight loss [21]. 

Dietary fibre (DF) has also received much attention due to its potential in weight regulation and is commonly used as an ingredient in food supplements or nutraceuticals for weight control. Epidemiological evidence supports DF consumption in reducing the incidence of overweight and obesity [22]. However, according to intervention studies, DF supplementation has facilitated weight loss in some cases but minor or null effects have been observed in others [23]. It is widely known that DF prolongs gastric emptying and retards the absorption of nutrients in the small intestine [24]. Among other effects, the prolonged presence of nutrients in the intestine influences the release of peptide hormones involved in appetite/satiety regulation [25]. Moreover, the longer mastication of foods rich in DF, which requires both time and effort, prolongs oral exposure time and raises satiety signals [22]. These effects largely depend on the physicochemical characteristics of the DF, which are often not reported in DF publications. Several authors support fibre viscosity as the main characteristic that determines appetite and satiating effects [26,27]. Thus, viscous DF, such as beta-glucans (BG), pectins and gums induce thickening when mixed with liquids, but the degree of thickening depends on their chemical composition, structure, concentration, and molecular weight [28]. In a study carried out with beverages containing oat BG, it was shown that reducing the natural viscosity of BG induced higher postprandial CCK, GLP-1, and PYY responses, and lowered ghrelin concentrations. These effects were partially reflected in the subjective ratings of appetite and satiety [29]. The authors attributed the reduced peptide levels to the viscosity delaying and preventing close interaction between nutrients and the gastrointestinal mucosa, required for the stimulation of enteroendocrine cells and satiety peptide production. In contrast, there was a significant dose–response in another study, with a positive correlation between the grams of oat BG and PYY postprandial area under the curve, with the effects on PYY mediated by viscosity and BG concentration [30]. To date, there is a broad number of human studies that suggest oat BG increases the perception of appetite [26] and satiety [22], most being acute studies. Other authors propose studying over longer periods of time (the whole day) in acute studies in order to better understand the effects of BG on satiety [30,31]. However, the effects of the regular consumption of oat BG have been less studied, and in some cases no differences in appetite hormones and ratings have been observed [22]. Considering that supplements for weight control are taken on a regular basis, it is relevant to assess their potential effect on appetite/satiety not only in acute consumption since adaptation to prolonged exposures might influence their effects.

Considering the aforementioned factors, a randomised, crossover study was carried out in overweight subjects to assess the influence of the acute and regular consumption of decaffeinated green coffee phenolic (GC) and decaffeinated GC combined with oat BG (GC/BG) nutraceuticals on appetite and satiety, analysed subjectively through VAS, and on food intake, in order to evaluate if the nutraceuticals (GC and GC/BG) could ultimately lead to weight loss. Moreover, in a subgroup of volunteers, the acute and regular effects of the nutraceuticals on appetite and satiety were validated objectively through analysing relevant related hormones. 

## 2. Materials and Methods

### 2.1. Subjects

Twenty-nine volunteerswith overweight and obesity (17 male/12 female, mean age 45.24 ± 1.77, range 28–59 years with body mass index (BMI) 30.09 ± 0.64 kg/m^2^) participated in this study. The recruitment was carried out by placing advertisements at the Institute of Food Science, Technology and Nutrition (ICTAN-CSIC), at Complutense University of Madrid campus (UCM), at health centres, and also through social networks. Although it was not an objective of the present work to study the effects of gender on the appetite and satiety response, we aimed for gender parity during recruitment. Inclusion criteria were men and women, 18–60 years old with BMI 25–35 kg/m^2^. Exclusion criteria were suffering chronic pathology other than overweight, obesity or pre-diabetes, smoking, vegetarianism, pregnancy in women, and taking dietary supplements or hormones. 

The study was approved by the Clinical Research Ethics Committee of Hospital Universitario Puerta de Hierro, Majadahonda in Madrid (Spain) and the Bioethics Committee of Consejo Superior de Investigaciones Científicas (CSIC). It also followed the guidelines laid down in the Declaration of Helsinki for experiments in humans. Written informed consent was obtained from all subjects. The study was registered in Clinical Trials (NCT05009615).

### 2.2. Nutraceutical

In the study, B-Can Oat Beta-Glucan with 70% concentration, molecular weight between 100–200 kg/mol and density of 0.4–0.5 g/mL, provided by Garuda International, Inc. (Exeter, CA, USA: data on molecular weight and density are according to suppliers’ information) was used. Its content in soluble and insoluble DF, physicochemical properties (swelling, water-holding capacity, and oil-holding capacity) and viscosity were measured in our institute, as described in Mateos et al. [32]. The decaffeinated green coffee extract used to produce the GC and GC/BG nutraceuticals was provided by PharmaFoods S.L. (Barcelona, Spain), and its phenolic composition was analysed by our team [32]. Decaffeinated green coffee was used because we aimed to focus on the effects of phenolic compounds in green coffee on obesity; we aimed to assess nutraceutical products that can be consumed by the largest group of people possible; one of the comorbidities associated with obesity is hypertension, thus it is appropriate to avoid administering caffeine to people with overweight and obesity; by using decaffeinated green coffee, we could use higher amounts of coffee without having to take into account caffeine sensitivity in the volunteers. Nutraceutical formulation containing GC/BG extracts was designed so that volunteers consumed 5 g/d of BG with 70% concentration, and GC provided 600 mg/d PP. In a previous dose–response study carried out in our group [32], this formulation showed to be the most effective in counteracting obesity-related comorbidities. Nutraceuticals were sealed in individual sachets that contained half of the daily dose, and labelled A or B for blinding.

### 2.3. Study Design

The study was a randomised, cross-over, blind trial that lasted 20 weeks. Volunteers were randomly assigned to begin with the nutraceutical that contained GC (15 volunteers) or GC/BG (14 volunteers) extracts in the first intervention, which was 8 weeks long. After the first intervention, they continued with a 4-week washout period, and then consumed the other nutraceutical they had not previously consumed for another 8 weeks. When we established the subgroups that would start consuming GC or GC/BG, about half were females and the other half were males. In both interventions, volunteers had to take the nutraceutical twice a day, half an hour before breakfast and lunch, dissolved in 250 mL of water. On the day before each visit, participants had to avoid consuming caffeine and PP rich foods. 

**Figure 1:**
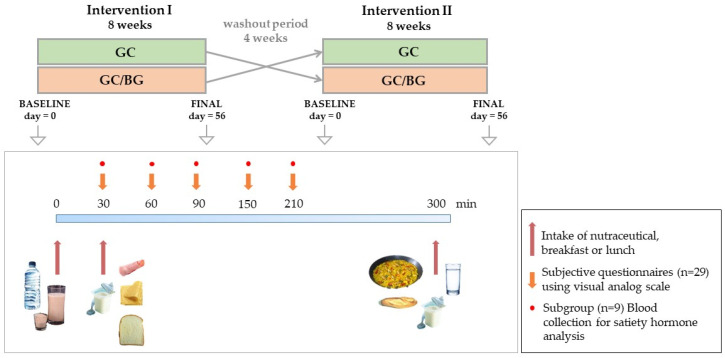
Study design and interventions carried out at each study visit.

At the baseline (day = 0) and end (day = 56) of each intervention stage, participants attended the Human Nutrition Unit (HNU) in ICTAN after a 12-h fast and their body weight was measured on a Tanita BC-418 MA device. Then, after consuming the corresponding nutraceutical, satiety was estimated by visual analog scales (VAS) and through measurement of food intake. Additionally, in a subgroup of volunteers (*n* = 9), blood samples were taken at different times to analyse the concentration of satiety hormones cholecystokinin (CCK), peptide-YY (PYY), glucagon like peptide-1 (GLP-1), ghrelin, and leptin in order to carry out objective measurements of appetite and satiety. The study design is detailed in Figure 1.

### 2.4. Subjective Satiety and Food Intake Measurements. Dietary Assessment

On the days volunteers attended the HNU after an overnight fast, they were invited to take the nutraceutical (GC or GC/BG) (time = 0 min) dissolved in 250 mL of water. Thirty minutes later, they were offered a standardized polyphenol-free breakfast (time = 30 min) consisting of yogurt, ham, cheese, and white bread, which they could eat *ad libitum* (detailed in Table 1). Immediately before breakfast and after nutraceutical consumption, volunteers were asked to fill in a questionnaire about their sensations of appetite and satiety at different times (30, 60, 90, 150, and 210 min). They were expected to answer the following questions: ‘How satiated do you feel?’, ‘How hungry are you?’, ‘How great is your desire to eat?’, and ‘How much food could you eat?’. The volunteers had to express their answers using visual analog scales (VAS) that consisted of a 100 mm horizontal line with a minimum (0 points) and a maximum (10 points) in their ends. On the 0–10 scale, answers were graded from 0 = no sensation of satiety, hunger or desire to eat, up to 10 = strong sensation of satiety, hunger or desire to eat. Regarding the question ‘How much food could you eat?’, the answer represented 0 = no food at all, up to 10 = a lot of food.

Five hours after consuming the nutraceutical (time = 300 min; Figure 1), they were invited to have a standardized lunch, which consisted of paella, bread (90 g), and yogurt (125 g). The paella was provided by a restaurant that cooks meals for CSIC workers. The cook agreed to follow a paella recipe in which vegetables usually included (onions, green and red peppers, and tomato), were avoided in order to reduce the amount of polyphenols in the meal, and otherwise having common ingredients, including chicken and seafood. The initial amount of paella provided to the volunteers was a large portion (750 g), so that volunteers could eat *ad libitum* until they were comfortably full and satisfied. All the non-consumed food was accurately weighed. Energy intake and macronutrient composition of the breakfast and lunch (Table 1) were calculated using DIAL software for Windows [version 3.0.0.5; Department of Nutrition, School of Pharmacy (UCM) and Alceingeniería, S.A. Madrid, Spain]. 

Volunteers were asked to keep their dietary habits, physical activity, and lifestyle unchanged during the intervention trial. However, in order to control possible changes, they filled in 72-h dietary records the week before each visit, i.e., the week before the start of the interventions (baseline values) and the week before the end of the intervention with GC and GC/BG (final values). In each stage, physical activity was monitored during the same week using accelerometers.

### 2.5. Objective Satiety Evaluation: Hormone Measurements

The group of 9 volunteers who agreed to participate in this part of the intervention completed the subjective questionnaires and ate breakfast and lunch as the rest of volunteers. Before consuming the nutraceutical, a nurse inserted a canula in the antecubital vein of the non-prevailing arm. Blood samples were collected at 30, 60, 90, 150, and 210 min after consuming the nutraceutical using tubes without anticoagulant or EDTA-coated to obtain serum and plasma samples, respectively. After centrifugation at 3000× *g* for 10 min at 4 °C, samples were aliquoted and stored at −80 °C until further analysis.

GLP-1, ghrelin and leptin concentrations were analysed in plasma using the Bio-Rad Multiplex Diabetes kit. The analytes were measured in duplicate on a Bio-Plex MAGPIX™ Multiplex reader connected to a Bio-Plex ProTM Wash Station. Software Bio-Plex Manager™ MP (Luminex Corporation, Austin, TX, USA) was used for data processing. Results were expressed as pg/mL plasma. Serum PYY and CCK were measured using Enzyme-Linked Immunosorbent (ELISA) kits (Cloud-Clone Corp, Katy, TX, USA) following protocols provided by the supplier. Results were expressed as pg/mL serum.

### 2.6. Statistical Analysis

To estimate sample size in the subjective satiety study, we considered body weight as the main variable and that it was a randomized, cross-over intervention. In addition, the statistical power of 80%, a level of statistical significance of 5%, a standard deviation of 6.5 were assumed, and we aimed at estimating a difference of 2.5. Considering all these premises, 38 subjects were expected to be recruited. With respect to the objective satiety study, we considered similar studies, such as the work carried out by Boix-Castejón et al. [2], where the primary outcome was detecting changes in satiety-related peptides after 2 months following the intake of a phenolic extract. A sample size of 8 participants was established.

Statistical analysis was carried out using IBM SPSS statistics 25 software. A linear mixed model was used for statistical analysis of energy, macronutrient and micronutrient intake data recorded from reports 72-h prior to each visit to the HNU, as well as for body weight measurements. With this analysis, the nutraceutical intake order was taken into account. In addition to studying the values of each variable at day 0 and day 56 of each intervention, the rate of change [(final value − baseline value)/baseline value] were determined to better estimate any effects produced by the nutraceuticals, that is, how much the variable increased or decreased with respect to the baseline value. 

According to PYY, CCK, GLP-1, ghrelin, and leptin results, the total area under the curve (AUC) from 30 to 210 min was estimated using the linear trapezoidal rule. In addition, the maximum concentration (C_MAX_) and time to maximum concentration (T_MAX_) were also calculated. The following comparisons were then made using the non-parametric Wilcoxon’s signed rank sum test: the acute effects of GC versus GC/BG on day 0 at the different time points, GC versus GC/BG on day 56 at the different time points and the effects of regularly consuming GC (day = 0 vs. day = 56) and GC/BG (day = 0 vs. day = 56) on AUC, C_MAX_, T_MAX,_ and satiety-related hormone levels. Moreover, in the 29 volunteers, the same comparisons were made for food intake and subjective satiety measurements, again using the non-parametric Wilcoxon’s signed rank sum test. The level of statistical significance was set at 0.05. Results were expressed as mean ± standard error of the mean (S.E.M) unless specified otherwise.

## 3. Results

### 3.1. Subjective Satiety/Appetite and Food Intake Measurements

**Figure 2:**
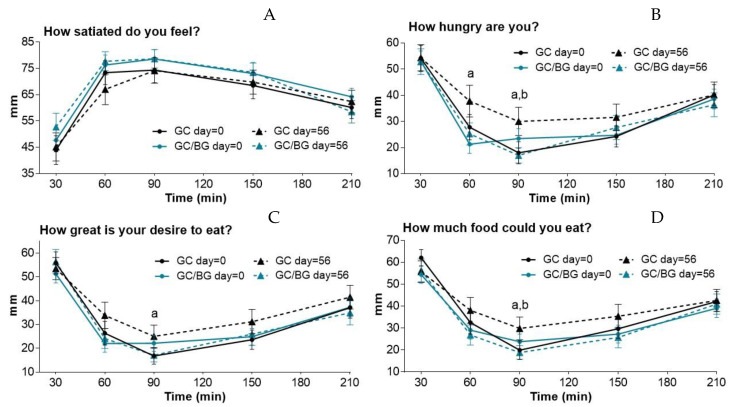
Variations of satiety and appetite sensations registered by the volunteers from 30 to 210 min after GC and GC/BG consumption on day = 0 and day = 56. Values correspond to the means ± SEM of scores (*n* = 29) recorded by VAS. Significant differences according to Wilcoxon’s signed rank sum test: (**A**) no significant differences. (**B**) ^a^ *p* < 0.05 GC day = 0 compared with GC day = 56; ^b^ *p* < 0.05 GC day = 56 compared with GC/BG day = 56. (**C**) ^a^ *p* < 0.01 GC day = 0 compared with GC day = 56. (**D**) ^a^ *p* < 0.05 GC day = 0 compared with GC day = 56; ^b^ *p* < 0.05 GC day = 56 compared with GC/BG day = 56.

Subjective appetite score variations from 30 to 210 min after GC or GC/BG extract consumption at the beginning (day = 0) and at the end (day = 56) of each intervention are represented in Figure 2. According to VAS scores, satiety and appetite sensations did not change throughout the study with either of the nutraceuticals. However, few differences related to hunger were found depending on time and the product. After GC consumption, hunger sensation was significantly higher at 60 min (*p* = 0.028) and 90 min (*p* = 0.004) on day = 56 than on day = 0 at the same times. Desire to eat (‘How great is your desire to eat?’ and ‘How much food could you eat?’) was also significantly higher 90 min after GC consumption on day = 56 than on day = 0 (*p* = 0.006 and *p* = 0.011, respectively). No differences were found in the acute studies with the GC/BG carried out at baseline and after consuming this nutraceutical for 8 weeks (final values).

The comparison between both nutraceuticals showed that hunger sensation was significantly lower 90 min after GC/BG consumption than after GC consumption on day 56 (*p* = 0.029). A reduced desire to eat (‘How much food could you eat?’) was also observed 90 min (*p* = 0.03) after GC/BG consumption compared with GC. No differences between GC and GC/BG were found on day 0.

Food intake at breakfast (30 min) and lunch (300 min) after consumption of GC or GC/BG products showed no significant differences at day 0 of the study nor after consuming the nutraceutical extracts for 8 weeks (day = 56).

### 3.2. Body Weight. Dietary Assessment. Physical Activity

According to the 72-h dietary reports, volunteers kept their dietary habits unchanged during the 20-week study (Table 2). At the baseline of each intervention (day = 0), before consuming GC or GC/BG, there were no differences in energy or macro and micronutrient intake. Similarly, at the end of the interventions (day = 56), after regular GC and GC/BG consumption, there were no differences in the dietary parameters measured. Moreover, the rate of change of energy or nutrient intake, in each intervention showed no statistically significant modifications. Therefore, neither GC nor GC/BG produced changes in dietary habits. Accordingly, the volunteers did not show changes in body weight measurements along the study (Table 2). According to the FAO/WHO/UNU Expert Consultation Report on Energy and Protein Requirements (1985), their physical activity was moderate and did not show modifications (data not shown).

### 3.3. Hormone Analysis

**Figure 3:**
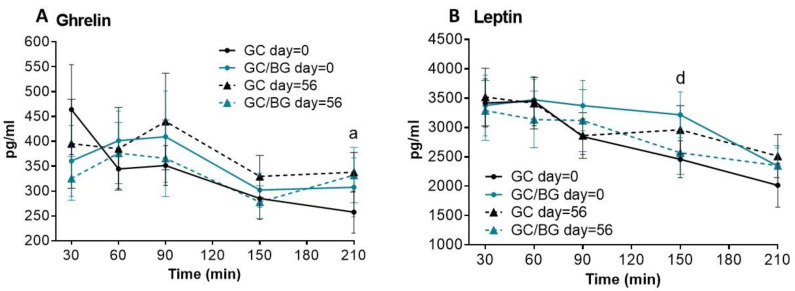
Plasma ghrelin and leptin concentrations from 30 to 210 min after GC and GC/BG consumption on day = 0 and day = 56. Values correspond to the means ± SEM, *n* = 9. Significant differences according to Wilcoxon’s signed rank sum test: (**A**) ^a^ *p* < 0.05 GC day = 0 compared with GC day = 56. (**B**) ^d^ *p* < 0.05 GC day = 0 compared with GC/BG day = 0.

CCK, PYY, GLP-1, ghrelin, and leptin concentrations after the consumption of GC or GC/BG extracts at each time point studied (30, 60, 90, 120, 150, 180, and 210 min) corresponding to day 0 and day 56 are recorded in Table 3. Moreover, variations of plasma ghrelin and leptin concentrations after consumption of the nutraceuticals studied are shown in Figure 3. Plasma ghrelin concentrations were significantly higher (+31.1%, *p* = 0.027) at 210 min after GC consumption on day = 56 compared to day = 0. No other statistical differences were observed in the rest of appetite/satiety-related hormones analysed after 8 weeks consuming both nutraceuticals compared to baseline. When the effects of the two nutraceuticals were compared on day 0, only higher plasma leptin levels (+30.9%, *p* = 0.025) at 150 min after GC/BG consumption were observed compared to GC consumption. 

Nutrikinetic results (AUC, C_MAX,_ and T_MAX_) are represented in Table 4. After consumption of the nutraceuticals, no differences were observed on CCK, PYY, GLP-1, ghrelin, and leptin AUC_30–210_. However, ghrelin T_MAX_ was delayed significantly (+1.2 h, *p* = 0.030) after GC consumption on day = 56 compared with day 0, and ghrelin C_MAX_ increased significantly (+21.3%, *p* = 0.002) after GC consumption compared with GC/BG on day 56.

## 4. Discussion

This study was aimed at understanding the effects of the acute and sustained consumption of a decaffeinated GC extract based nutraceutical on appetite and satiety in people with overweight and obesity. In addition, it investigated the acute and sustained effects of a novel nutraceutical, which consists of the combination of decaffeinated GC with an oat BG extract, with known satiating properties, in order to investigate possible synergistic effects on appetite and satiety. To our knowledge, the joined effects of phenols in green coffee and oat BG have not been studied before and could present potential as a tool to lose weight, among other beneficial health effects, considering the individual properties of these food components.

Appetite, hunger, satiety, and fullness are subjective parameters not easy to quantify [3]. The review by Stuby et al. [3] states that it is not clear if the four terms had the same meaning in the studies included, and in some cases, they were analysed as four different outcomes, whereas in others they were used synonymously. Clear and consistent definitions of these terms are essential in science to make progress in this area. In laypeople, such as the volunteers, this inconvenience is greater. In addition, most studies that measure these parameters use questionnaires and line scales, such as the VAS, to express results, as in the present study. However, this method has several limitations. Regarding the VAS, extreme values usually are avoided, the distances between units do not reflect perceptual distances, etc. Additionally, appetite, hunger, satiety, and fullness sensations differ among people. It is not clear if everybody has the same perception of hunger, and hunger intensity expressed on a scale differs. Moreover, circadian rhythms should not be disregarded, thus the time point of assessment is relevant. Taking into account all these restrictions, the present VAS results showed that volunteers (*n* = 29) described greater hunger sensation after regularly consuming GC (day = 56), compared to the end of the intervention with GC/BG (day = 56). This result could be attributed to the oat β-glucan component in GC/BG, as it likely prolonged the presence of nutrients in the intestine, which affected the release of peptide hormones involved in appetite/satiety regulation [25]. The oat β-glucan extract used in this study presented a relatively high concentration but low viscosity, thus the interaction with the gastrointestinal mucosa, required for the stimulation of enteroendocrine cells and satiety peptide production, could be favoured [29]. Moreover, since GC/BG was consumed twice a day for 8 weeks, appetite/satiety peptides would remain at the higher levels for longer. Furthermore, short-chain fatty acids produced from colonic fermentation of the oat BG may have also activated the release of peptides involved in appetite regulation [22]. All the aforementioned mechanisms may have contributed to the results observed in the present objective satiety intervention (Table 3).

On the one hand, focusing on the satiety/hunger peptides that showed changes in the objective study when GC was compared to GC/BG on day 0, leptin levels were higher after GC/BG consumption at 150 min versus GC. Slightly higher leptin concentration was also observed at 90 and 210 min, to a lower extent at 150 min, pointing to higher satiety at the later time points (between 90–210 min); this outcome contrasts with subjective results, which did not show differences in either hunger or satiety sensation between GC and GC/BG on day 0. According to Hassanzadeh-Rostami and Faghih [33], soluble or insoluble dietary fibre intake seem to not produce changes in leptin values in the short term but may reduce leptin levels in the long term in people with obesity. Regarding the present study, it seems that BG may increase leptin levels in the short term as well. Leptin is a lipostatic hormone secreted by adipose tissue that plays a crucial role in the central regulation of food intake and energy homeostasis at multiple levels so that its net effect within the hypothalamus is to inhibit food intake and to increase energy expenditure [11]. On the other hand, after regular consumption of the nutraceuticals, ghrelin C_MAX_ was lower with GC/BG than GC, which points to lower hunger after regularly consuming the new nutraceutical (GC/BG). Ghrelin is a gastrointestinal hormone synthesized in the fundus of the stomach that stimulates food intake and thus plays a role in the satiety cascade. In fact, it has been proposed as a biomarker of satiety in the short and long-term [34,35]. This outcome is in agreement with the lower subjective hunger results that the participants described on day 56 at 1.5 h after consuming GC/BG. Therefore, according to ghrelin results, there is accordance between objective and subjective hunger results. Thus, the outcome of the present study does not support ghrelin playing a doubtful role in the regulation of satiety in humans because of the non-association between its postprandial responses and postprandial subjective satiety [36]. With respect to GC/BG, the DF extract component of the new nutraceutical may have contributed to the decrease in ghrelin levels as β-glucan enriched bread reduced plasma ghrelin and peptide YY concentrations in the short term [31]; however, to our knowledge, the effects of regular consumption of oat β-glucans on ghrelin have not been described before. Nevertheless, the differences in observed ghrelin and leptin concentrations did not lead to changes in food intake in the subgroup of nine volunteers, nor were any differences observed in food intake in the 29 volunteers. 

According to VAS results, an outstanding result in the present study was that participants (*n* = 29) described greater hunger sensation after regularly consuming GC (day = 56) compared to acute GC consumption (day = 0). Similar results were observed in the subgroup of nine participants (data not shown). This outcome could be associated with the delay in ghrelin T_MAX_ (from 0.4 h to 1.6 h) observed after regular GC consumption for 8 weeks compared to acute consumption. This is the first human trial that comparatively approaches the acute and the chronic effects of a green coffee phenolic extract on hunger, thus should be further studied. As aforementioned, the effects of long-term consumption of PP rich food on hunger and satiety have been studied less than the acute effects. Hussein et al. [37] observed an increase in blood leptin and GLP-1 levels in mice after chronic consumption of mate tea, with similar phenolic composition to green coffee [38], which was associated with decreased food intake and body weight in the animals. When the same authors [37] investigated the effects of the major constituents of mate, the acute administration of hydroxycinnamates, also present in GC [32], increased GLP-1, which may be related to the modulation of leptin levels, not observed in the present study. However, these results should be taken with caution as there are discrepancies between rodent and human findings regarding reduction in energy intake and body weight [39]. It is important to note that satiety is a complex mechanism, mediated by multiple signals that are integrated into the hypothalamus. In addition, fat reduction and metabolic normalization also play a role in regulating these peptides due to the variation of adiposity [2]. Accordingly, in the aforementioned study with hibiscus and lemon verberena extracts, the increased GLP-1, normalized ghrelin, and decreased leptin concentration in overweight subjects was also associated with a decrease in adiposity, and weight loss may be accompanied by less secretion of leptin by adipocytes [2]. In sum, the subjective appetite/hunger responses and the objective hormone analysis are in agreement according to ghrelin results but not to leptin. However, the changes observed did not translate into differences in food intake at lunch. In addition to the lack of differences in food intake induced by the nutraceuticals, there were no modifications in body weight throughout the study. To the latter outcome has contributed there being no changes in the participants’ dietary habits nor physical activity throughout the study. Regarding the other objective satiety measurements studied (Table 3), there were no differences in the PYY, CCK, and GLP-1 at any of the time points studied on day 0 or day 56 with both nutraceuticals. According to these parameters, there is a certain disagreement between objective and subjective assessments as the volunteers did not describe differences in satiety, but they did for appetite sensations. This outcome is in line with previous studies, which also observed that postprandial physiological responses of satiety-related gut hormones are not necessarily reflected in the subjective appetite sensation [29,36]. It is well known that leptin may enhance the suppressive effects of gut peptides and stimulate GLP-1 release from L-cells [40]; however, this effect was not observed in the present study, as the leptin changes previously described did not lead to modifications in GLP-1 levels. The results here observed are in agreement with a randomized cross-over trial where the acute ingestion of decaffeinated coffee and chlorogenic acid did not affect GLP-1 concentration [41], and with an acute study with instant caffeinated or decaffeinated coffee that did not show effects on satiety [18].

Limitations included the inconveniences described in the discussion regarding subjective satiety assessments, although the studies were always carried out at the same time under similar conditions. The main dish at lunch, paella, was cooked on each study day, so there may be differences in palatability due to the cooking that might have affected its consumption by volunteers. This meal was chosen because it is well accepted by Spanish people; however, it is possible that some volunteers did not enjoy it as much as the others. The study carried out in 29 subjects was slightly underrepresented.

## 5. Conclusions

The new nutraceutical consisting of the combination of an oat β-glucan extract with a decaffeined phenolic green coffee extract appears to increase satiety and reduce appetite, which is related with higher levels of leptin (acute effect) and lower ghrelin levels (sustained effect), respectively, in addition to lower hunger sensation. Therefore, the acute and regular consumption of the new GC/BG nutraceutical may play a role in helping to maintain body weight. In contrast, weight management properties of GC seem to not be consistent in modifying appetite/satiety related peptides and the sustained effects of GC on these parameters should be further studied.

## Figures and Tables

**Table 1:** Macronutrient composition (g) and energy value (Kcal) of breakfast and lunch.

	Proteins	Carbohydrates	Fats	Dietary Fibre	Energy Value (Kcal)
**Standarized breakfast**					
Natural yogurt, 250 g	10	14	7	0	154
Sugar, 16 g	0	16	0	0	64
Gouda cheese, 20 g	5	0	5	0	60
Ham, 60 g	18	6	9	0	182
White bread, 50 g	4	25	2	2	136
**Standardized lunch**					
Paella, 750 g	26	173	39	3	1162
White bread, 90 g	8	46	1	3	235
Yogurt, 125 g	5	7	3	0	77

**Table 2:** Energy, macronutrient, and micronutrient intakes recorded in 72-h reports filled in by volunteers before each study visit, and body weight measurements.

	GC	GC/BG	*p*		GC	GC/BG	*p*
**Energy (Kcal)**				**PUFA (g)**			
Baseline	2019 ± 100	2023 ± 112	N.S.	Baseline	12.4 ± 1.1	12.8 ± 1.2	N.S.
Final	2115 ± 118	2128 ± 115	N.S.	Final	11.9 ± 0.9	11.8 ± 0.7	N.S.
Rate of change	8.3 ± 5.7	8.5 ± 5.6	N.S.	Rate of change	1.9 ± 9.0	10.4 ± 8.0	N.S.
**Protein (g)**				**PUFA/SFA**			
Baseline	82.8 ± 4.1	79.7 ± 3.6	N.S.	Baseline	0.50 ± 0.04	0.48 ± 0.04	N.S.
Final	82.6 ± 4.7	86.9 ± 4.5	N.S.	Final	0.42 ± 0.03	0.46 ± 0.04	N.S.
Rate of change	5.4 ± 7.1	12.2 ± 6.1	N.S.	Rate of change	3.9 ± 9.6	4.0 ± 8.2	N.S.
**CHO (g)**				**[PUFA + MUFA]/SFA**			
Baseline	199 ± 11	205 ± 14	N.S.	Baseline	1.8 ± 0.1	1.8 ± 0.1	N.S.
Final	204 ± 11	200 ± 13	N.S.	Final	1.9 ± 0.1	1.9 ± 0.1	N.S.
Rate of change	9.8 ± 7.2	6.3 ± 8.7	N.S.	Rate of change	14.0 ± 8.3	15.1 ± 8.3	N.S.
**Fibre (g)**				**Cholesterol (mg)**			
Baseline	24.0 ± 1.7	23.8 ± 1.7	N.S.	Baseline	316 ± 25	331 ± 23	N.S.
Final	22.1 ± 1.9	22.2 ± 1.3	N.S.	Final	295 ± 28	359 ± 34	N.S.
Rate of change	−5.5 ± 8.3	2.8 ± 5.8	N.S.	Rate of change	6.4 ± 10.8	25.4 ± 15.0	N.S.
**Fat (g)**				**Calcium (mg)**			
Baseline	84.7 ± 5.8	86.5 ± 6.3	N.S.	Baseline	743 ± 42	14.3 ± 0.8	N.S.
Final	93.6 ± 6.8	96.9 ± 7.2	N.S.	Final	703 ± 61	16.0 ± 1.0	N.S.
Rate of change	16.4 ± 7.9	18.0 ± 7.6	N.S.	Rate of change	−1.3 ± 6.4	12.2 ± 6.6	N.S.
**SFA (g)**				**Iron (mg)**			
Baseline	28.0 ± 2.2	28.5 ± 2.0	N.S.	Baseline	13.6 ± 0.7	86.4 ± 2.4	N.S.
Final	30.9 ± 2.8	31.5 ± 2.7	N.S.	Final	13.4 ± 0.7	86.8 ± 2.4	N.S.
Rate of change	18.2 ± 10.0	19.3 ± 10.4	N.S.	Rate of change	11.1 ± 9.4	0.4 ± 0.3	N.S.
**MUFA (g)**				**BW (Kg)**			
Baseline	34.5 ± 2.4	34.7 ± 2.2	N.S.	Baseline	86.8 ± 2.4	86.4 ± 2.4	N.S.
Final	41.9 ± 3.2	42.8 ± 3.2	N.S.	Final	86.4 ± 2.3	86.8 ± 2.4	N.S.
Rate of change	30.9 ± 10.0	29.3 ± 8.9	N.S.	Rate of change	−0.4 ± 0.3	0.4 ± 0.3	N.S.

Values represent mean ± SEM, *n* = 29. *p* corresponds to GC day = 0 values compared with GC/BG day = 0 values, GC day = 56 values compared with GC/BG day = 56 values and GC rate of change compared with GC/BG rate of change based on a linear mixed model analysis. Rate of change represents [(final value−baseline value)/baseline value]. Carbohydrate (CHO); saturated fatty acid (SFA), monounsaturated fatty acid (MUFA); polyunsaturated fatty acid (PUFA); body weight (BW).

**Table 3:** Effects of the consumption of GC or GC/BG nutraceuticals on satiety-related hormones.

		GC					GC/BG					
pg/mL	Time (min)	30	60	90	150	210	30	60	90	150	210	*p* *^1^
**PYY**	**Day = 0**	1615 ± 275	1543 ± 277	1528 ± 277	1532 ± 289	1542 ± 284	1636 ± 282	1584 ± 274	1557 ± 259	1577 ± 293	1568 ± 286	N.S.
	**Day = 56**	1638 ± 298	1593 ± 298	1564 ± 304	1537 ± 290	1539 ± 300	1620 ± 281	1591 ± 287	1531 ± 268	1509 ± 256	1527 ± 293	N.S.
	*p* *^2^	N.S.	N.S.	N.S.	N.S.	N.S.	N.S.	N.S.	N.S.	N.S.	N.S.	
**CCK**	**Day = 0**	566 ± 65	550 ± 42	549 ± 47	541 ± 55	535 ± 50	576 ± 48	557 ± 49	552 ± 44	589 ± 49	552 ± 45	N.S.
	**Day = 56**	581 ± 56	570 ± 48	591 ± 51	531 ± 45	557 ± 60	593 ± 50	582 ± 49	582 ± 48	576 ± 57	570 ± 62	N.S.
	*p* *^2^	N.S.	N.S.	N.S.	N.S.	N.S.	N.S.	N.S.	N.S.	N.S.	N.S.	
**Ghrelin**	**Day = 0**	464 ± 91	344 ± 40	351 ± 40	285 ± 42	258 ± 42	361 ± 71	401 ± 60	409 ± 92	302 ± 35	308 ± 59	N.S.
	**Day = 56**	395 ± 89	385 ± 83	440 ± 97	330 ± 42	338 ± 40	325 ± 44	376 ± 62	366 ± 77	278 ± 33	332 ± 56	N.S.
	*p* *^2^	N.S.	N.S.	N.S.	N.S.	0.027	N.S.	N.S.	N.S.	N.S.	N.S.	
**Leptin**	**Day = 0**	3419 ± 409	3446 ± 414	2844 ± 303	2458 ^a^ ± 315	2016 ± 374	3375 ± 517	3471 ± 345	3372 ± 427	3216 ^b^ ± 390	2341 ± 308	0.025
	**Day = 56**	3518 ± 491	3414 ± 440	2863 ± 388	2961 ± 411	2512 ± 367	3290 ± 510	3136 ± 481	3117 ± 528	2568 ± 366	2350 ± 339	N.S.
	*p* *^2^	N.S.	N.S.	N.S.	N.S.	N.S.	N.S.	N.S.	N.S.	N.S.	N.S.	
**GLP-1**	**Day = 0**	208 ± 20	200 ± 24	222 ± 26	194 ± 18	176 ± 18	165 ± 21	199 ± 16	208 ± 23	201 ± 13	165 ± 14	N.S.
	**Day = 56**	174 ± 17	178 ± 15	209 ± 20	226 ± 18	192 ± 19	191 ± 20	183 ± 22	213 ± 24	197 ± 28	203 ± 23	N.S.
	*p* *^2^	N.S.	N.S.	N.S.	N.S.	N.S.	N.S.	N.S.	N.S.	N.S.	N.S.	

Values represent mean ± SEM, *n* = 9. PYY = peptide tyrosine–tyrosine, CCK = Cholecystokinin, ghrelin, leptin, GLP-1 = glucagon like peptide-1 levels are expressed in pg/mL *p* *^1^ corresponds to GC compared with GC/BG at each time point on day = 0 and on day = 56. *p* *^2^ corresponds to day = 0 compared to day = 56 at each time point with GC and GC/BG. Superscripts (^a,b^) correspond to significant differences according to Wilcoxon’s signed rank sum test

**Table 4:** Effects of the consumption of GC or GC/BG extracts in the area under the curve 30–120 min (AUC_30–120_), maximum concentration (C_MAX_) and time to maximum concentration (T_MAX_) for the satiety-related hormones studied.

		AUC (pg/mL*h)				C_MAX_ (pg/mL)			T_MAX_ (h)		
		GC	GC/BG		*p* AUC	GC	GC/BG	*p* C_MAX_	GC	GC/BG	*p* T_MAX_
**PYY**	**Day = 0**	4624 ± 843	4730 ± 835		N.S.	1644 ± 283	1673 ± 280	N.S.	0.7 ± 0.4	0.8 ± 0.3	N.S.
	**Day = 56**	4686 ± 888	4621 ± 815		N.S.	1664 ± 295	1636 ± 282	N.S.	0.2 ± 0.1	0.3 ± 0.1	N.S.
	*p* *^2^	N.S.	N.S.			N.S.	N.S.		N.S.	N.S.	
**CCK**	**Day = 0**	1637 ± 146	1701 ± 137		N.S.	617 ± 63	609 ± 46	N.S.	0.8 ± 0.3	0.8 ± 0.3	N.S.
	**Day = 56**	1682 ± 147	1736 ± 159		N.S.	615 ± 58	616 ± 54	N.S.	0.7 ± 0.3	1.2 ± 0.4	N.S.
	*p* *^2^	N.S.	N.S.			N.S.	N.S.		N.S.	N.S.	
**Ghrelin**	**Day = 0**	966 ± 117	1054 ± 175		N.S.	501 ± 85	471 ± 80	N.S.	0.4 ± 0.2	0.6 ± 0.1	N.S.
	**Day = 56**	1120 ± 182	988 ± 153		N.S.	494 ± 88	407 ± 75	0.038	1.6 ± 0.3	1.4 ± 0.4	N.S.
	*p* *^2^	N.S.		N.S.		N.S.	N.S.		0.03	N.S.	
**Leptin**	**Day = 0**	8177 ± 937	9495 ± 994		N.S.	3699 ± 411	4176 ± 417	N.S.	0.2 ± 0.1	1.0 ± 0.3	N.S.
	**Day = 56**	8950 ± 1072	8472 ± 1165		N.S.	3779 ± 480	3769 ± 555	N.S.	0.7 ± 0.4	0.5 ± 0.3	N.S.
	*p* *^2^	N.S.		N.S.		N.S.	N.S.		N.S.	N.S.	
**GLP-1**	**Day = 0**	601 ± 53	580 ± 41		N.S.	250 ± 18	232 ± 15	N.S.	0.8 ± 0.3	1.0 ± 0.2	N.S.
	**Day = 56**	612 ± 47	598 ± 69		N.S.	241 ± 18	234 ± 22	N.S.	1.7 ± 0.2	1.4 ± 0.3	N.S.
	*p* *^2^	N.S.		N.S.		N.S.	N.S.		N.S.	N.S.	

Values represent mean ± SEM, *n* = 9. CCK = Cholecystokinin, PYY = peptide tyrosine–tyrosine and GLP-1 = glucagon like peptide-1, *p* AUC, *p* T_MAX_ and *p* C_MAX_ correspond to GC compared with GC/BG on day = 0 and on day = 56 according to Wilcoxon’s signed rank sum test. *p* *^2^ corresponds to day = 0 compared to day = 56 with GC and with GC/BG according to Wilcoxon’s signed rank sum test.

## Data Availability

Data has not been deposited in any repository, yet it can be made available to researchers upon request.
